# The impact of altering restaurant and menu option position on food selected from an experimental food delivery platform: a randomised controlled trial

**DOI:** 10.1186/s12966-023-01456-8

**Published:** 2023-05-19

**Authors:** Filippo Bianchi, Madison Luick, Lauren Bandy, Jonathan Bone, Stefan Kelly, James Farrington, Jovita Leung, Abigail Mottershow, Filip Murar, Susan A. Jebb, Hugo Harper, Rachel Pechey

**Affiliations:** 1 Behavioural Insights Team, London, UK; 2 Nuffield Department of Primary Care Health Sciences, University of Oxford, Oxford, UK; 3 Nesta, London, UK

**Keywords:** Repositioning, Food delivery platform, Energy content, RCT

## Abstract

**Background:**

Overconsumption is one of the most serious public health challenges in the UK and has been linked to increased consumption of food ordered through delivery platforms. This study tested whether repositioning foods and/or restaurant options in a simulated food delivery platform could help to reduce the energy content of users’ shopping basket.

**Methods:**

UK adult food delivery platform users (N = 9,003) selected a meal in a simulated platform. Participants were randomly allocated to a control condition (choices listed randomly) or to one of four intervention groups, (1) food options listed in ascending order of energy content, (2) restaurant options listed in ascending order of average energy content per main meal, (3) interventions 1 and 2 combined (4) interventions 1 and 2 combined, but food and restaurant options repositioned based on a kcal/price index to display options lower in energy but higher in price at the top. Gamma regressions assessed the impact of interventions on total energy content of baskets at checkout.

**Results:**

The energy content of participants’ baskets in the control condition was 1382 kcals. All interventions significantly reduced energy content of baskets: Compared to control, repositioning both foods and restaurants purely based on energy content of options resulted in the greatest effect (-209kcal; 95%CIs: -248,-168), followed by repositioning restaurants (-161kcal; 95%CIs: -201,-121), repositioning restaurants and foods based on a kcal/price index (-117kcals; 95%CI: -158,-74) and repositioning foods based on energy content (-88kcals; 95%CI: -130,-45). All interventions reduced the basket price compared to the control, except for the intervention repositioning restaurants and foods based on a kcal/price index, which increased the basket price.

**Conclusions:**

This proof-of-concept study suggests repositioning lower-energy options more prominently may encourage lower energy food choices in online delivery platforms and can be implemented in a sustainable business model.

**Supplementary Information:**

The online version contains supplementary material available at 10.1186/s12966-023-01456-8.

## Background

Overweight and obesity contribute to poor health globally [1]. According to Public Health England the additional burden of disease created by overweight and obesity in 2019 resulted in an estimated cost of £6.1 billion to the NHS. Reducing energy intake is a key component to obesity prevention [2].

Analyses of major UK restaurant chains showed that only 9% of dishes had an energy content of less than 600 kcals a meal, and 47% of dishes were at least 1000 kcals or more, which equates to half of the daily-recommended energy intake for a woman [3]. Similar analyses of more discretionary food items on offer at restaurants, including starters, side dishes, and desserts, found over a fifth of foods in each category exceeded 600 kcal [4]. Out-of-home consumption of meals from restaurants, cafes and takeaways, and in particular, consumption of takeaway or delivery food, have been associated with higher energy intake and higher BMI. [5, 6]. Given the increasing use of delivery platforms, this is an important target for population-based interventions [7, 8].

The environments in which food is purchased influence our behaviours. Automatic reactions to how options are presented to us in our environment, sometimes lead to choices and behaviours that may detrimentally affect our health [9]. By changing the environment in which food is selected or purchased, it may be possible to increase the likelihood of selecting healthier foods through unconscious mechanisms and without restricting choice. Systematic reviews have identified that changing the positioning of food in physical environments can influence purchasing patterns [10, 11]. Similar patterns were seen in studies conducted in simulated online supermarkets, where ranking products in order of increasing saturated fat or energy content resulted in less saturated fat and less energy selected by participants [12, 13].

Studies looking at the impact of such choice architecture interventions in restaurant-type settings have found that increasing availability of healthy foods, promotion and pricing changes were all effective in changing purchasing decisions [10, 11, 14–16]. Additionally, similar interventions, which repositioned food items in supermarket settings (e.g. placing or removing items from end of aisles, changing which items were next to each other), found this could affect purchasing patterns, although with mixed effects [17–20]. However, little is known about how choice architecture health promoting interventions affect behaviour in the context of food delivery platforms.

This proof-of-concept study explores how interventions repositioning lower-energy options to be more prominent influence food choices in a simulated food delivery platform and if these interventions can help to reduce the amount of energy selected.

## Methods

### Design

This was a five-arm randomised controlled trial. Participants were recruited to obtain a UK representative sample, with specific quotas set for age, gender, location, and income. Quotas were set based on data from the UK Office for National Statistics [21–23]. Participants were individually randomised to one of the five trial arms.

### Interventions

**Fig. 1:**
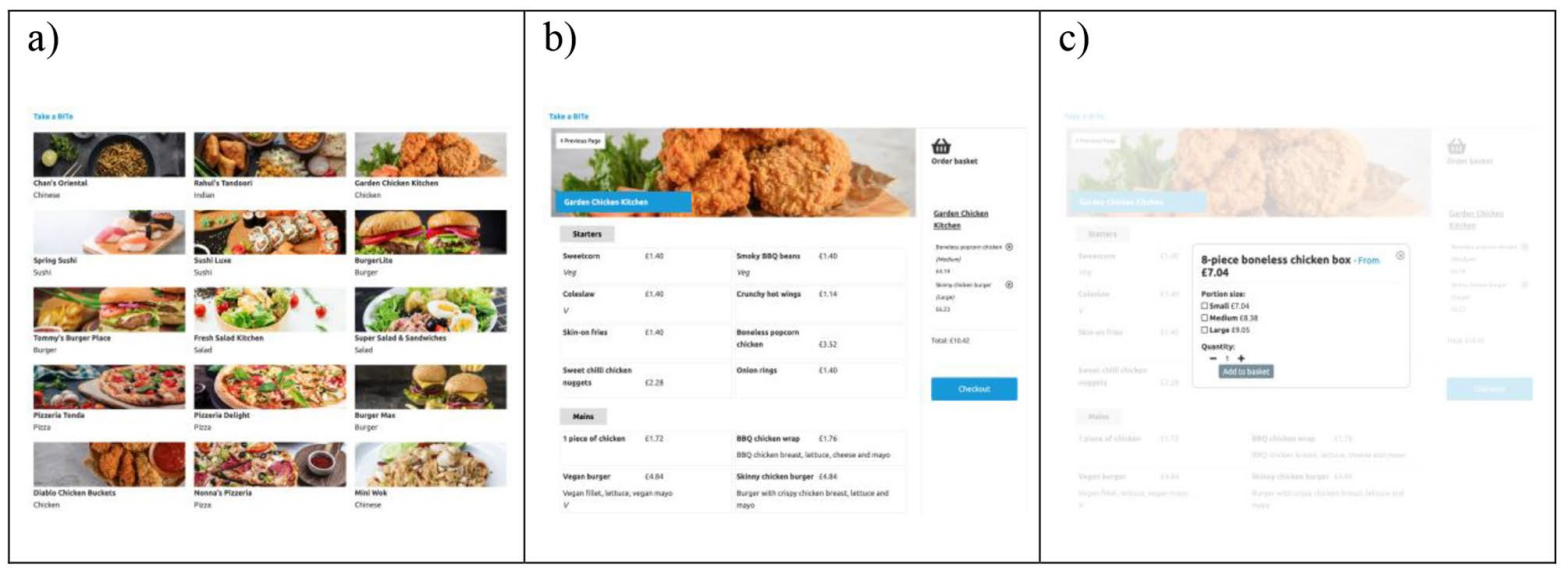
Screenshots of the 'Take a BITe’ online platform, showing (**a**) a subsection of the restaurant selection page, (**b**) a sample food menu, and (**c**) a portion size pop-up window

This study was conducted using ‘Take a BITe’, a simulated food delivery platform created and hosted by the Behavioural Insights Team. The platform has a similar design to real-world food delivery platforms such as Deliveroo, UberEats, and JustEat. There were 21 restaurants and 570 food or drink items, each in three different portions (i.e. 1710 individual food options in total). Data were used from research on the nutritional content of takeaway food options [24]. On average, mains on *‘Take a BITe’* contain 840 kcal and cost £8.60 (examples of the platform are shown in Fig. 1).

**Fig. 2:**
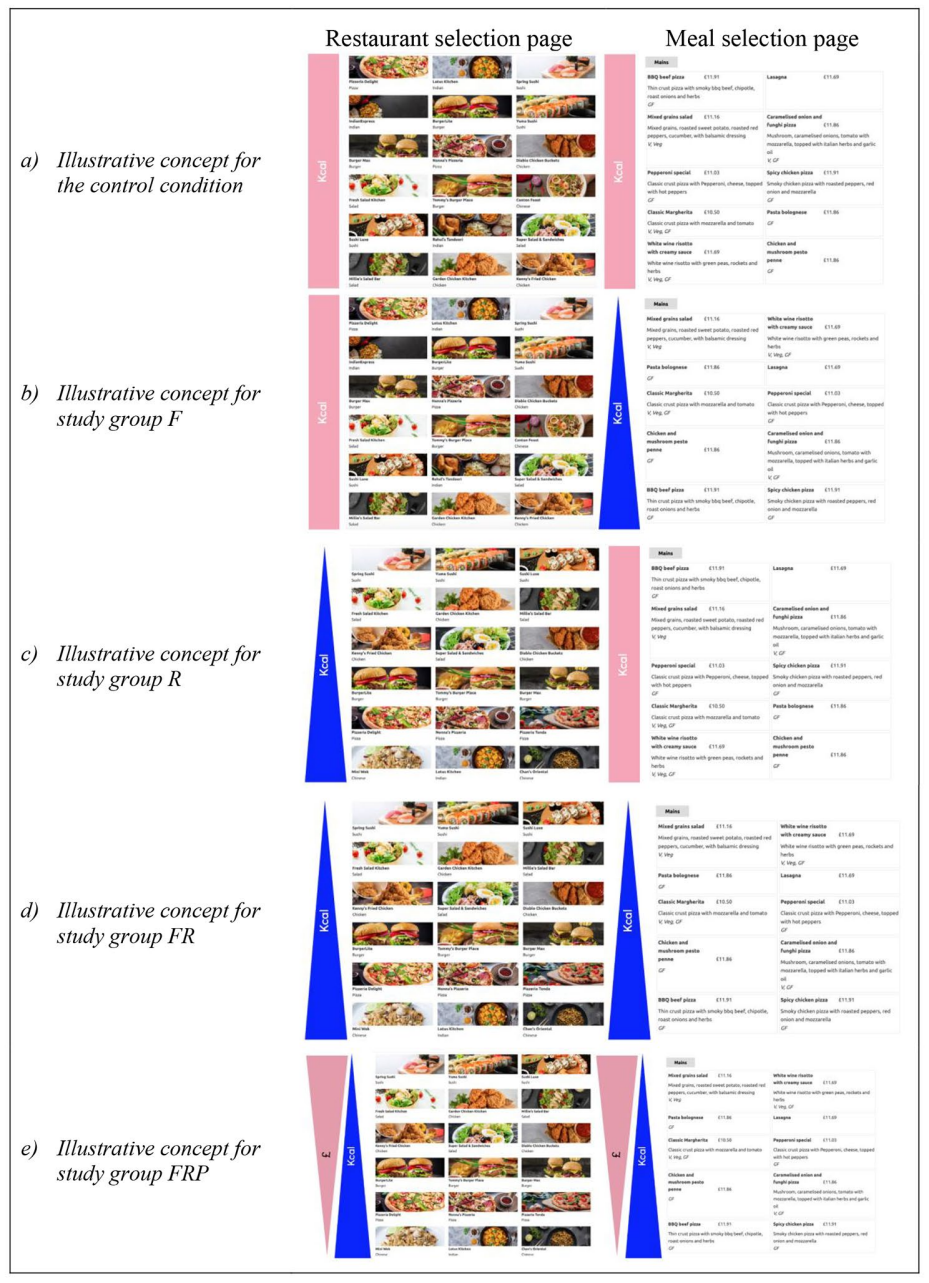
Illustrative concept diagrams of the study conditions showing (**a**) control, (**b**) foods only, (**c**) restaurants only, (**d**) foods and restaurants only, and (e) foods and restaurants plus price

The five study conditions (shown illustratively in Fig. 2) were:

1. Control (C): restaurants and foods positioned in a random order. Food items were randomly ordered within food categories, but the order in which food categories appear was not randomised (e.g. starters presented first, followed by mains, etc.).
2. Foods only (F): all food items listed in ascending order of energy content within each restaurant and food category (e.g. starters, mains), with restaurants ordered at random.
3. Restaurants only (R): restaurants listed in ascending order of average energy content per main meal, with foods listed at random within restaurant menus.
4. Foods and restaurants (FR): a combination of interventions 1 and 2, where foods were listed in ascending order of energy content in menus and restaurants were listed in ascending order of average energy content per main meal.
5. Foods and restaurants plus price (FRP): restaurants and foods were repositioned as in intervention 3, but this time using a kcal-price index. For the restaurant order, the index was calculated by dividing the average energy content of mains by the average price of mains for each restaurant. For the food order within food categories of restaurant menus, the index was calculated by dividing the energy of a dish by the price. This meant that lower energy but higher price options were positioned at the top. The FRP trial arm primarily sought to test for proof-of-concept if positioning based on consideration for both health and profits, with price here used as a proxy for profits, could impact energy purchased while remaining sensitive to financial constraints of industry.

### Participant recruitment

Participants were required to be users of a food delivery platform and adults (18 years or older) living in the UK. Our study aimed to collect data from 9,000 participants in order to be powered to detect a 65 kcal reduction in total energy, with a standard deviation estimated at 550 kcal, 80% power, 5% significance level, and corrections for multiple comparisons. 65 kcal was determined based on findings from a previously conducted study [25].

### Procedure

Participants were recruited via the Behavioural Insights Team’s online recruitment platform, Predictiv, which connects with a research panel aggregator. After eligibility and screening, participants were randomised to one of five study conditions. On the simulated delivery platform, participants were given the following instructions:

*Imagine you are using our online delivery platform to order food for a single meal for yourself. You can use our food delivery platform just like you would in real life: you can browse through multiple restaurants, view their menus, and add or remove foods from your basket. Once you are happy with your order you can click ‘checkout’ to complete the task.*

From here, participants were directed to the online delivery platform and completed the task, with foods and restaurants displayed in accordance with their randomised condition.

Participants received no set budget. They did not pay any money nor did they receive the food items they ordered. Participants were also asked questions about their age, gender, ethnicity, region, income, education level, height, weight, socioeconomic status, and the frequency with which they order from food delivery platforms. Data from these questions was then used for secondary analyses and as covariates in the models. Participants were given a small financial incentive by the panel provider upon completion of the study.

### Ethics

Ethics approval was granted for the study protocol by the Central University Research Ethics Committee (Ref: R65010/007). The protocol was pre-registered on the Open Science Framework (https://osf.io/cp7kv).

### Statistics

After recruitment, participants were excluded from analysis if they had duplicate identifiers (with the first record kept), if they dropped out from the experiment, or if their basket contained less than 150 kcal or more than 4000 kcal at checkout, since these were pre-specified as unlikely values. There were also attention checks during the study, and if a participant failed these they were excluded. Attention checks involved asking the participants to respond to a survey question. The first attention check was the following:

*People are very busy these days and many do not have time to pay close attention to what they are reading. We are testing whether people read questions. To show that you’ve read this much, answer both “Extremely interested” & “Very interested”.*

If participants failed to select the correct responses, they were given a second attention check as follows:

*You didn’t select the correct answers to our last question. Your attention to the survey questions is very important for our research, so we’d like to give you another chance to respond. To show that you are paying attention, answer both “Extremely interested” and “Very interested.“*

Primary analyses: The primary outcome was energy (kcal) in the basket at checkout. Gamma regression was used and all treatment groups were compared against the control and against each other. Estimated p-values were adjusted using the Benjamini-Hochberg procedure for 10 comparisons, and heteroscedasticity-robust HC3 standard errors were used for regression coefficients.

Secondary analyses: Secondary outcomes included the price of baskets, average energy content of mains on selected restaurant menus, and average energy content of standardised servings (the medium size) of selected main meal items. When analysing the average energy content of main meal dishes selected from restaurant menus, linear regression models were applied. Gamma regression was used for other outcomes and exploratory analyses, including the price of basket at checkout. P-values and standard errors were adjusted in the same way as for primary analyses.

Additional analyses used gamma regression models to explore potential variance in effects based on sex, socioeconomic position (SEP), BMI, and frequency of food delivery platform usage, and whether the study was taken on a mobile or desktop device. In these models, the participant characteristic was included as an interaction term with the intervention in the regression. Subgroup analyses used HC3 robust standard errors and estimated p-values using the Benjamini-Hochberg procedure for 10 comparisons.

In all models, participant characteristics, including SEP, sex, regional location, income, ethnicity, education, BMI, age, residence area, frequency of ordering on a delivery platform, device used for the study, time of day taking the survey, and day of the week taking the survey, were included to account for potential confounding variables. Gamma regression and linear regression were used respectively based on the expected skew of the outcome variables, with most expected to have a right-skew to which gamma regression was applied. Data were assessed visually to consider the skew of outcome variables and confirm the appropriate model was applied. R 4.1.3 was used for all analyses.

## Results

**Fig. 3:**
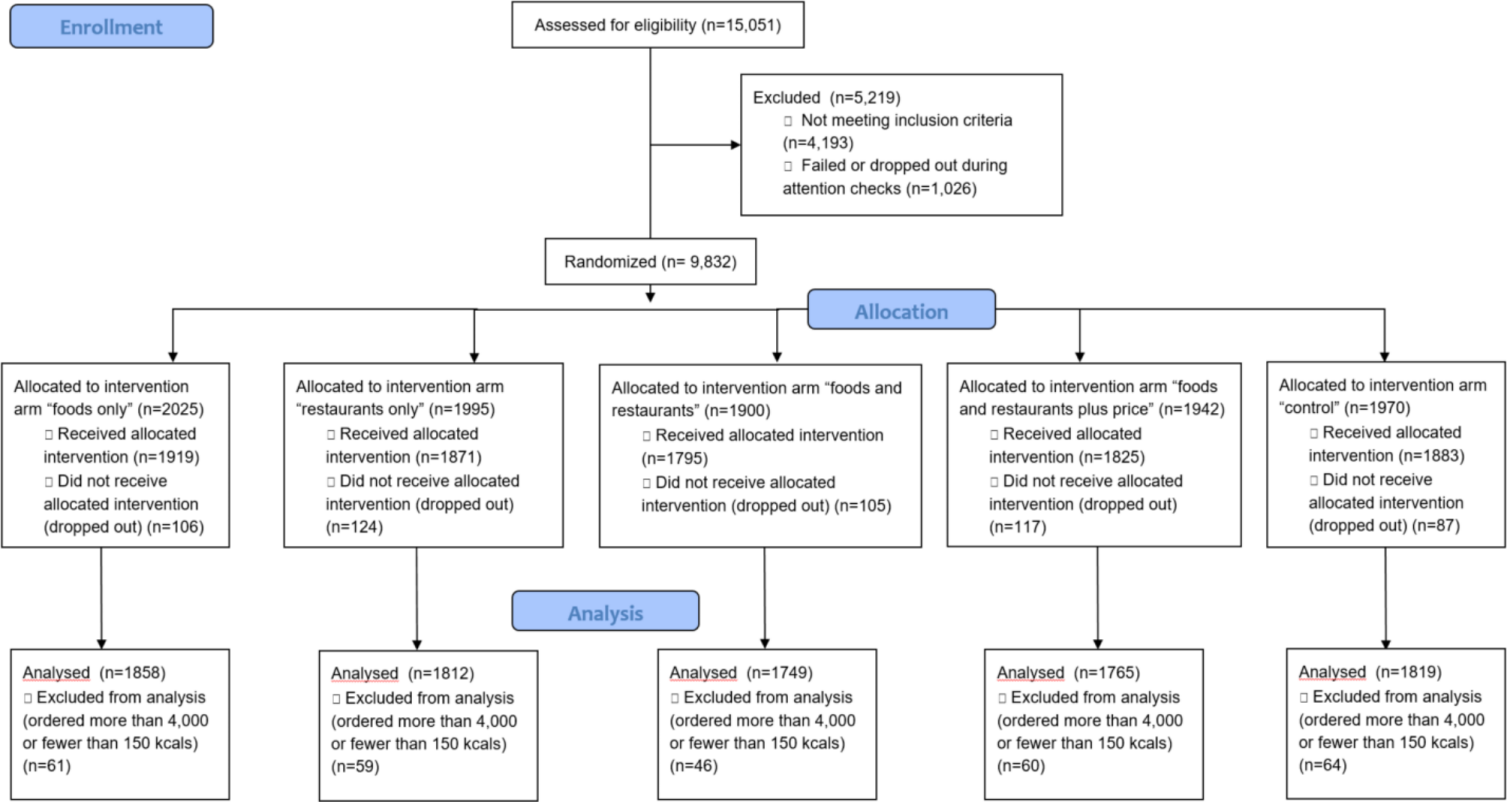
CONSORT flow diagram

The study was run from February to March 2022. 15,051 entrants were assessed for eligibility to participate. 5,148 were excluded for failing to meet inclusion criteria or failing attention checks. 9,293 completed the study task, but 290 were excluded from analysis because their hypothetical order baskets contained either more than 4,000 or fewer than 150 kcals (see CONSORT Flow Diagram, Fig. 3). The analysed sample contained 9,003 participants:

- 1819 in the control group,
- 1858 in the foods only repositioning intervention (F),
- 1812 in the restaurants only repositioning intervention (R),
- 1749 in the foods and restaurants repositioning intervention (FR), and.
- 1765 in the foods and restaurants repositioning based on energy content and price (FRP).

Across all conditions, participants ordered on average 1269 kcal with a value of £14.63 (see Supplementary Tables 1–2). Compared to the control, all four interventions significantly reduced the total energy content of participants’ baskets at checkout.

**Fig. 4:**
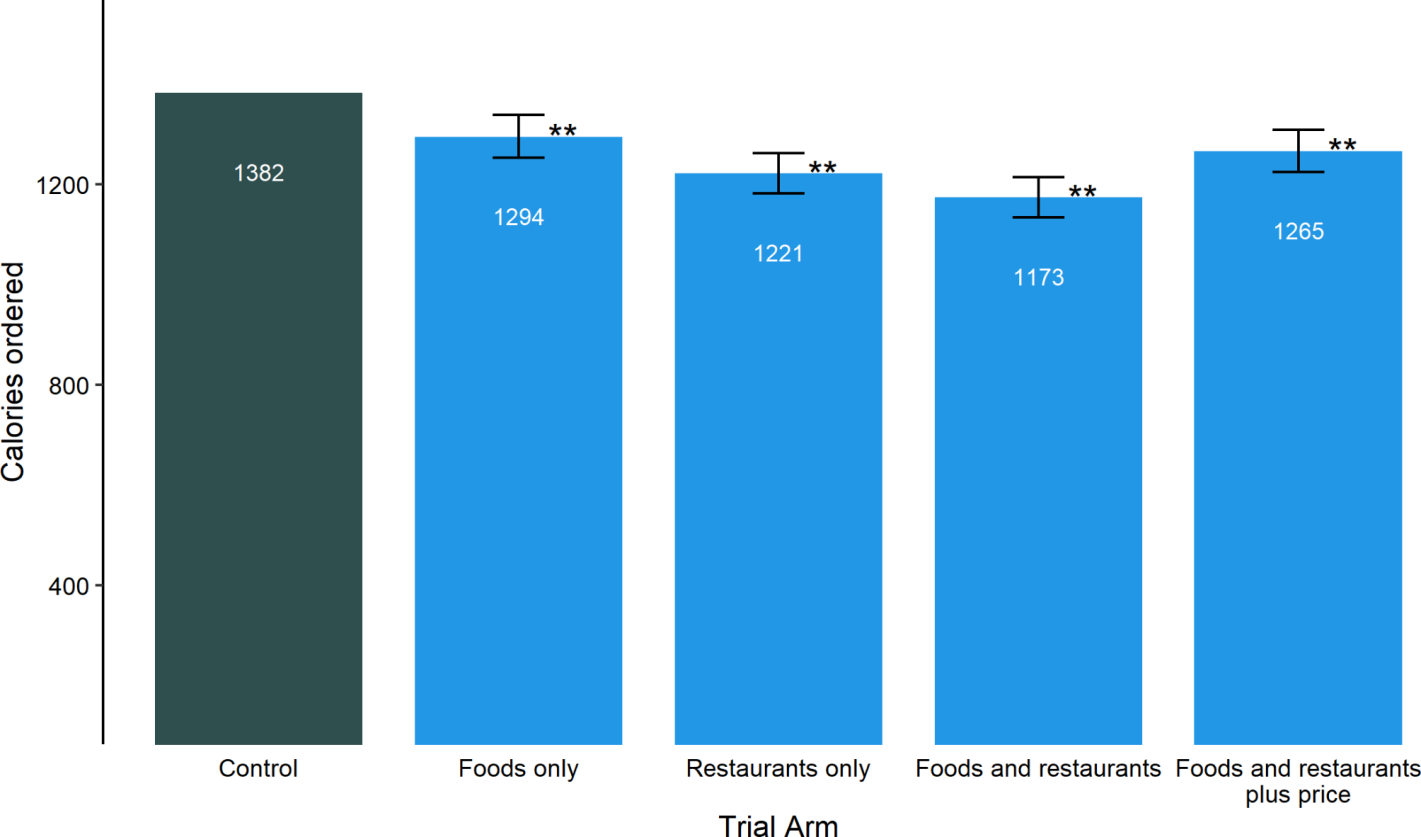
Mean energy from foods selected in the control group and the four intervention groups (foods repositioning, restaurants repositioning, and foods and restaurants repositioning, foods and restaurants plus price repositioning). Bar ranges represent 95% confidence interval, and ** indicates adjusted p < 0.01

Compared to the control, repositioning both foods and restaurants purely based on the energy content of options resulted in the greatest effect (-209 kcal; 95% CIs: -248, -168), followed by repositioning restaurants (-161 kcal; 95% CIs: -201, -121), repositioning restaurants and foods based on a kcal/price index (-117kcals; 95% CI: -158, -74) and repositioning foods based on their energy content (-88kcals; 95% CI: -130, -45) (Fig. 4; Supplementary Tables 3–4).

In a comparison of estimated marginal means for total energy in participants’ baskets, all interventions led to baskets with significantly lower energy contents than the control. All intervention groups significantly differed from each other in terms of their effect on the energy content of participants baskets, with the exception of interventions F (foods repositioning) and FRP (foods and restaurant plus price repositioning), which did not significantly differ (Table 1).

**Table 1:** Estimated percentage change between all study groups for total energy from foods selected. The reference group is the first group listed

Comparison (95% CI)	Foods only (F)	Restaurants only (R)	Foods and restaurants (FR)	Foods and restaurants plus price (FRP)
*Reference Group*				
Control	-6.8 (-10.4, − 3.3)	-13.2 (-17.1, -9.5)	-17.8 (-21.9, -13.9)	-9.2 (-13.0, -5.6)
F		-6.0 (-9.6, -2.5)	-10.3 (-14.1, -6.7)	-2.3 (-5.8, 1.1)
R			-4.1 (-7.7, -0.6)	3.5 (0.2, 6.7)
FR				7.3 (4.1, 10.4)

*Confidence intervals are calculated using HC3 heteroscedasticity-robust standard errors

The average energy content of mains and the energy density of a standardised serving was also lower in all interventions when compared to the control (Supplementary Tables 5–6).

**Fig. 5:**
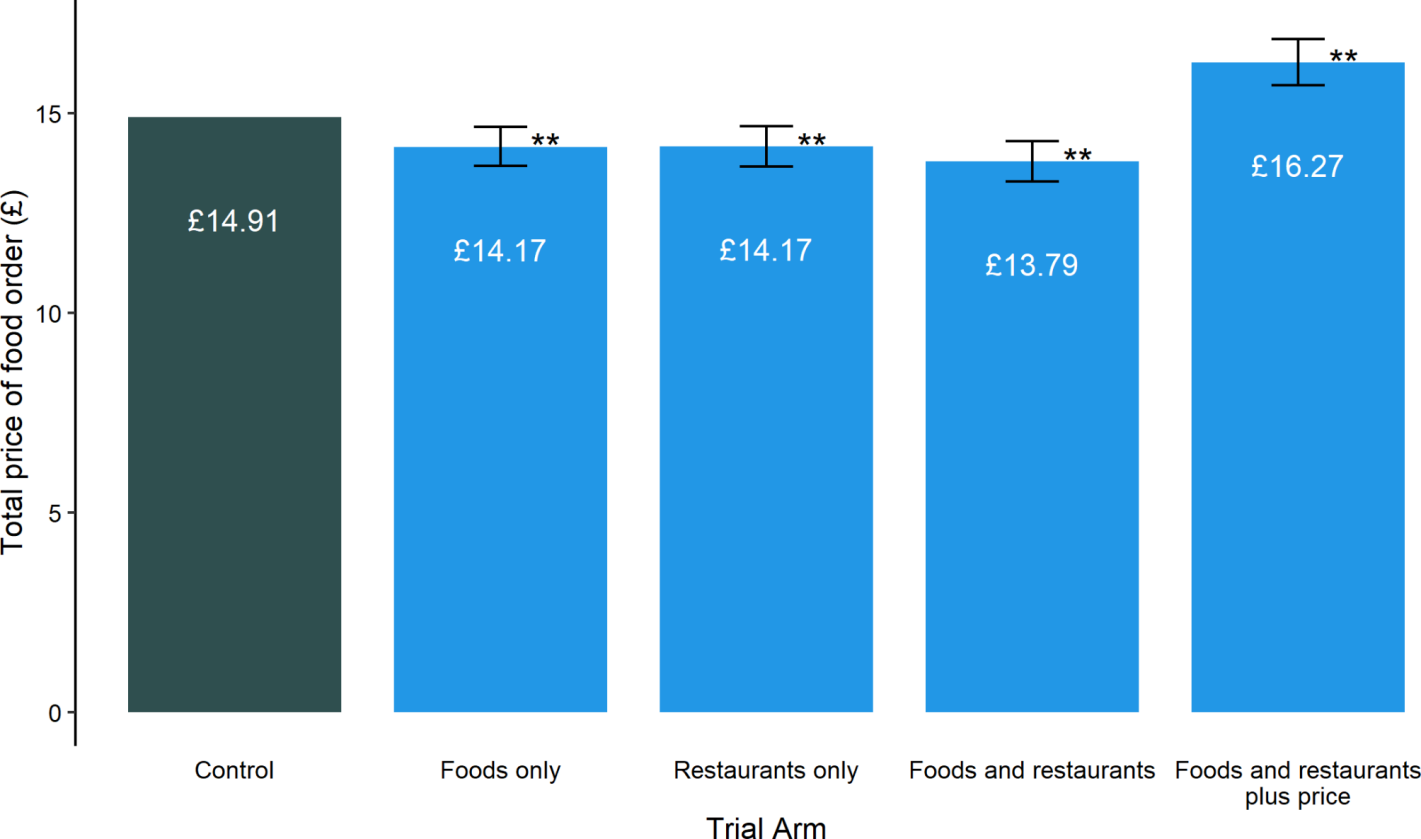
Mean money spent (£) in the control and four intervention groups. Bar ranges represent 95% confidence interval, and ** indicates adjusted p < 0.01

The total basket price at checkout varied between interventions. All interventions repositioning options purely based on the energy content of items reduced the basket price at checkout. However, the interventions that repositioned options to give more prominence to lower-energy but higher-price foods and restaurants (FRP) reduced the energy content of participants’ baskets whilst increasing the basket price (Fig. 5; Supplementary Table 7).

**Fig. 6:**
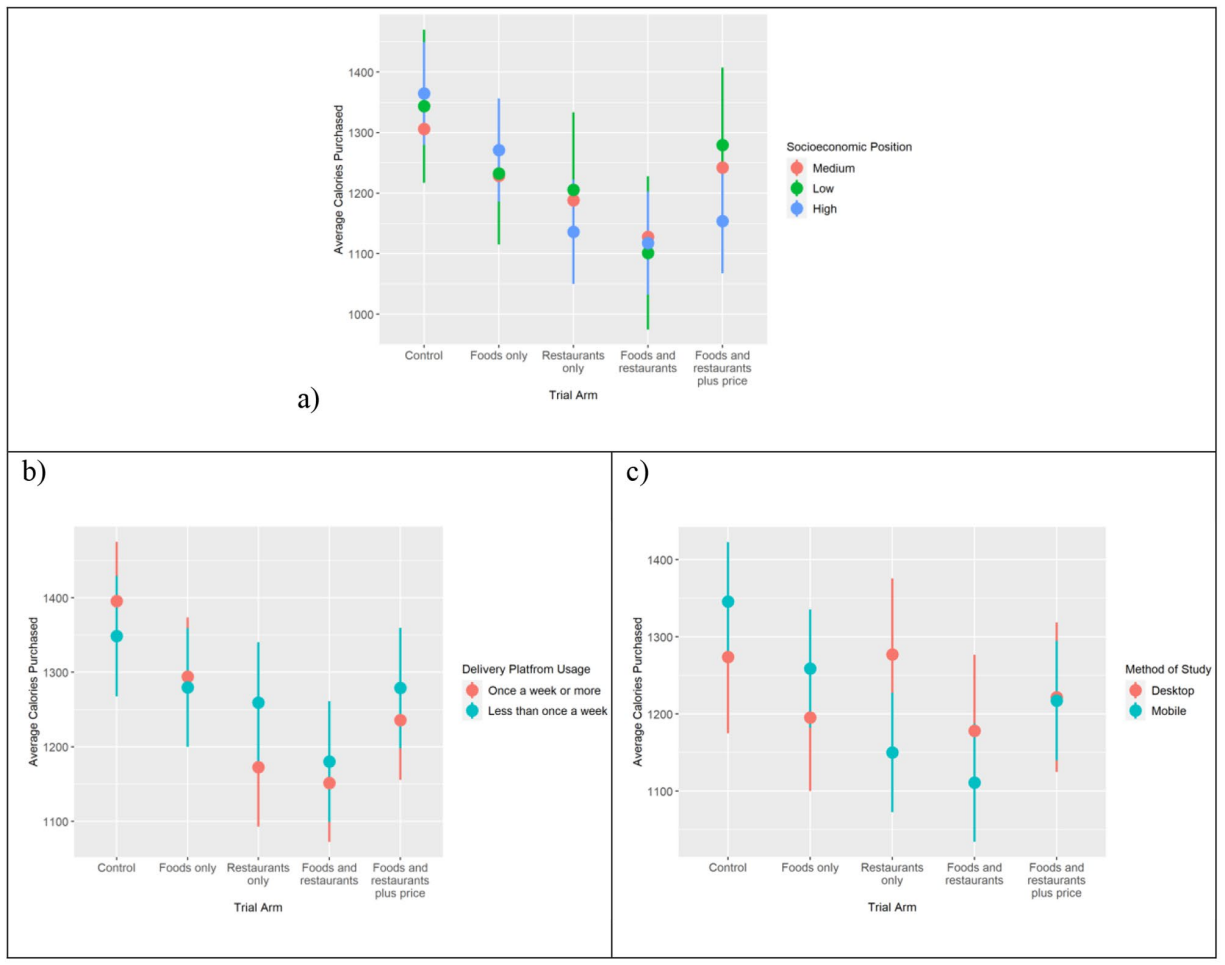
Marginal mean plots for average energy (in kcals) placed in basket by (**a**) socioeconomic position, (**b**) delivery platform usage, and (**c**) method of taking the study (mobile phone or desktop)

Subgroup analysis found there was a greater impact among people of higher versus medium SEP of the FRP intervention (147 fewer kcal ordered, 95% CI: 53–241, p-value: 0.022) (Fig. 6b, intervention FRP). See Supplementary Tables 8–12.

The study task could be undertaken from a desktop or mobile device. There was a greater impact of the intervention among people using a mobile device compared to desktop in the study conditions that involved repositioning restaurants purely based on energy content (199 fewer kcal ordered, p-value: 0.003 for study arm R;139 fewer kcal ordered, p-value: 0.046 for study arm FR) (Fig. 6b, intervention R and FR).

There was a greater intervention effect among participants that used a delivery platform more than once a week compared to those that used it less than once a week for the intervention repositioning restaurants based on the energy content of their mains (133 fewer kcal selected, p-value: 0.021 for study group R) (Fig. 6c).

All other subgroup analyses and interactions tested were not significant.

## Discussion

All the interventions tested in this study reduced the energy content of participants’ baskets, showing as a proof-of-concept that these approaches might have the potential to lower energy purchased from food delivery platforms. The greatest decrease was in the intervention that repositioned both foods and restaurants purely based on energy content. All interventions repositioning options purely based on energy content led to a decrease in total basket price, which may represent an implementation barrier for industry. An intervention designed to mitigate the potential reduction in revenue was successful in its aim, but less effective or no different in reducing the energy content of foods selected than the other price-agnostic interventions.

One of the strengths of this study is its relevance to real food purchasing platforms. The sample was large and broadly representative of the UK population and participants were free to choose a meal from a virtual site that closely mimicked online delivery platforms. The energy purchased in this study was comparable to energy content for takeaway food reported previously (medians for five takeaway food types of 1125–1820 kcal per meal) [24]. However, this study took place in a simulated environment and participants were not spending their own money and were not receiving the food they ordered, which may limit the extent to which the findings generalise to real-world environments. Additionally, in the control condition, foods were positioned within categories completely at random, whereas in real-world settings, ordering may be determined by other factors (e.g. popularity, sponsored preferences). While this study demonstrates the potential for the effects of ordering, the degree of impact in a particular setting may vary depending on the extent to which it alters the existing ordering. In previous studies where the effect of labelling and other supermarket interventions were tested in online experimental settings, it was found that the online studies had a greater effect size than findings from real-world purchasing, but the trend and pattern of results was generally the same [13, 26].

As a proof-of-concept, this study shows the potential impact of repositioning options in food delivery platforms to promote health outcomes and explores strategies to promote health outcomes whilst protecting the bottom line of restaurants and delivery platforms. Voluntary implementation in real-world settings may be challenging, as was previously seen when voluntary food reformulation targets resulted in limited changes [27], suggesting some regulation may be required for effective implementation. In the absence of existing regulation, we aimed to test whether changes in positioning could be implemented in a manner that also retained revenue, to increase the likelihood of voluntary adoption, but this was less effective in its primary aim to reduce energy intake.

This study focused on prominent positioning of items at the top of the page, based on evidence from previous research that primacy effects are a critical factor in boosting selection in online settings [13]. This study did not consider any long-term adaptations to delivery platform changes though it is possible that individuals will adapt to changes in their food environment, and learn to scroll further through the list to find favoured items.

It has been suggested that interventions focusing on changing the choice architecture in people’s environments may reduce disparities due to the universal application of interventions [9, 28]. Some of our findings are in line with this hypothesis. Specifically, there were no significant differences in the effects of interventions F, R, and FR between people from different SEP. However, for intervention FRP we found greater effects for people from higher versus lower SEP, possibly suggesting that these groups would have a greater benefit from these interventions, widening inequalities.

Post hoc we observed two other factors which may warrant further exploration. First, interventions that altered the restaurant position were more effective when participants accessed the study on a mobile phone compared to a desktop computer. This seems plausible since mobile device users may need to scroll further to see the same numbers of restaurants as on a desktop or laptop, and therefore be more likely to click on restaurants nearer the top. Second, the effect of interventions was smaller in the restaurants only repositioning conditions among participants who used food delivery platforms more often. One possible explanation for this is that frequent users may know more about how these platforms work, and more quickly search for what they want to select, rather than browsing.

## Conclusions

Repositioning of products and restaurants to make lower energy options more prominent in a simulated online delivery platform effectively reduced the amount of energy in recipients’ baskets. Although all interventions were effective, changing the order of both the foods and the restaurants was more effective than altering either alone. Further research is needed to better consider how to optimise these interventions and expand upon these proof-of-concept findings, but results indicate they could be implemented in a sustainable business model.

## Electronic supplementary material

Below is the link to the electronic supplementary material.

Supplementary Material 1

Supplementary Material 2

Supplementary Material 3

## Data Availability

Data will be made available upon request.

## List of Abbreviations

SEP: Socioeconomic position
BMI: Body mass index
