# Supplementary material for: The impact of altering restaurant and menu option position on food selected from an experimental food delivery platform: a randomised controlled trial
Source: Int J Behav Nutr Phys Act. 2023 May 19;20:60. doi: 10.1186/s12966-023-01456-8 (PMC10197857; doi:10.1186/s12966-023-01456-8)
Supplement: Supplementary file 3 — Supplementary Material 3 [file 12966_2023_1456_MOESM3_ESM.docx]

## Demographics Table

**Supplementary Table 1**. Proportion of respondents in each trial arm from demographic groups and mean energy purchased. Trial arms involve repositioning by: F (foods only), R (restaurants only), FR (foods and restaurants), and FRP (foods and restaurants plus price).

|  | **Control** | **F** | **R** | **FR** | **FRP** | **Total** | **Mean energy** |
| --- | --- | --- | --- | --- | --- | --- | --- |
| **Residence Area** |  |  |  |  |  |  |  |
| Rural | 19 | 19 | 19.6 | 21.2 | 20.7 | 19.9 | 1318.01 |
| Suburban | 48.7 | 49.4 | 47.5 | 46 | 46.7 | 47.7 | 1284.82 |
| Urban | 32.3 | 31.6 | 32.8 | 32.8 | 32.5 | 32.4 | 1216.38 |
| **Time of Day** |  |  |  |  |  |  |  |
| Morning - Breakfast | 10.4 | 11.1 | 11.7 | 11.4 | 10.7 | 11.1 | 1238.56 |
| Afternoon – Lunch | 31.8 | 31.2 | 31 | 32 | 30.3 | 31.2 | 1265.80 |
| Evening – Dinner | 28.5 | 26 | 27.3 | 26.6 | 27.9 | 27.3 | 1281.37 |
| Night | 29.3 | 31.7 | 30 | 29.9 | 31.2 | 30.4 | 1273.11 |
| **SEP Category** |  |  |  |  |  |  |  |
| Low | 7.8 | 9.5 | 7.8 | 8.2 | 7.8 | 8.2 | 1293.98 |
| High | 36.6 | 34.2 | 34.7 | 35.2 | 33.3 | 34.8 | 1253.61 |
| Medium | 55.6 | 56.2 | 57.6 | 56.7 | 58.9 | 57 | 1275.22 |
| **Frequency of Ordering** |  |  |  |  |  |  |  |
| Less than once a month | 21 | 23 | 19.9 | 20.6 | 21.3 | 21.2 | 1275.90 |
| A few times a week | 19 | 18.7 | 17.8 | 16.2 | 16 | 17.5 | 1241.50 |
| Every day | 1.9 | 1.6 | 2.2 | 1.8 | 2.7 | 2 | 966.17 |
| Once a month | 26.4 | 27.6 | 28.6 | 28.3 | 27.4 | 27.6 | 1303.71 |
| Once a week | 31.7 | 29.1 | 31.6 | 33.1 | 32.6 | 31.6 | 1269.54 |
| **Device Used** |  |  |  |  |  |  |  |
| Desktop | 18.3 | 21 | 18.6 | 19.6 | 19.9 | 19.5 | 1289.00 |
| Mobile | 81.7 | 79 | 81.4 | 80.4 | 80.1 | 80.5 | 1264.47 |
| **Location** |  |  |  |  |  |  |  |
| London | 13.9 | 12.8 | 12.3 | 13 | 12.6 | 12.9 | 1228.50 |
| Midlands | 16.7 | 17.9 | 17.1 | 16.7 | 17.3 | 17.1 | 1278.85 |
| North | 26.7 | 26.9 | 26 | 26.5 | 26.2 | 26.5 | 1261.26 |
| South and East | 27.2 | 28.8 | 28.7 | 29.2 | 30.2 | 28.8 | 1294.67 |
| Wales, Scotland & Northern Ireland | 15.5 | 13.6 | 15.9 | 14.6 | 13.8 | 14.7 | 1258.40 |
| **Income Category** |  |  |  |  |  |  |  |
| Less than £30,000 | 54.4 | 54.3 | 58.2 | 55.4 | 55.3 | 55.5 | 1256.87 |
| £30,000 and over | 45.6 | 45.7 | 41.8 | 44.6 | 44.7 | 44.5 | 1284.71 |
| **Gender** |  |  |  |  |  |  |  |
| Female | 50.9 | 51 | 49.4 | 51.1 | 50.9 | 50.6 | 1274.75 |
| Male | 47.8 | 47.7 | 49.2 | 47 | 47.8 | 47.9 | 1263.74 |
| Other gender | 1.4 | 1.3 | 1.3 | 1.9 | 1.4 | 1.5 | 1259.29 |
| **Ethnicity** |  |  |  |  |  |  |  |
| Asian | 5.8 | 6.8 | 5.8 | 6.2 | 6.2 | 6.2 | 1171.50 |
| Black | 3.9 | 3.3 | 4.7 | 4 | 4.3 | 4 | 1190.77 |
| Mixed | 2.6 | 2.4 | 2.5 | 3.5 | 2.9 | 2.8 | 1225.41 |
| Other | 0.7 | 0.7 | 0.3 | 1 | 0.7 | 0.7 | 1237.06 |
| White | 86.9 | 86.8 | 86.7 | 85.3 | 85.8 | 86.3 | 1281.59 |
| **Education Group** |  |  |  |  |  |  |  |
| High school completed | 53.7 | 54.5 | 53.9 | 53.6 | 54.4 | 54 | 1286.01 |
| Less than high school | 3 | 2.8 | 2.8 | 2.2 | 2.3 | 2.6 | 1300.32 |
| None of the above | 1 | 1.5 | 0.9 | 1.2 | 1.1 | 1.1 | 1276.68 |
| University degree | 42.3 | 41.2 | 42.4 | 43 | 42.2 | 42.2 | 1245.69 |
| **Day of the Week** |  |  |  |  |  |  |  |
| Sun | 8.2 | 8.6 | 8.2 | 8.7 | 8.8 | 8.5 | 1279.09 |
| Mon | 2.5 | 2.2 | 2 | 2.2 | 2 | 2.2 | 1329.77 |
| Tue | 42.4 | 41.6 | 41 | 42.9 | 42.2 | 42 | 1267.58 |
| Wed | 27.3 | 28.2 | 28.7 | 26.5 | 27.7 | 27.7 | 1259.38 |
| Thu | 15 | 13.6 | 14.4 | 14.8 | 13 | 14.2 | 1261.27 |
| Fri | 3.2 | 4 | 3.8 | 3 | 4.2 | 3.7 | 1351.59 |
| Sat | 1.3 | 1.9 | 1.8 | 1.8 | 2.1 | 1.8 | 1234.89 |
| **BMI Category** |  |  |  |  |  |  |  |
| Healthy weight | 42.3 | 42.7 | 44.6 | 43.7 | 44.4 | 43.5 | 1224.71 |
| Obese | 24.5 | 24.2 | 22.5 | 24.5 | 22.5 | 23.6 | 1336.43 |
| Overweight | 29.7 | 29.7 | 29 | 28.7 | 29.7 | 29.4 | 1291.63 |
| Underweight | 3.6 | 3.3 | 3.9 | 3.1 | 3.3 | 3.4 | 1179.90 |
| **Age Category** |  |  |  |  |  |  |  |
| Under 25 | 16.1 | 15.1 | 14.6 | 13.7 | 14.3 | 14.7 | 1192.03 |
| 25 to 54 | 60.3 | 59.5 | 60.2 | 62.5 | 62.5 | 61 | 1272.41 |
| 55 and over | 23.6 | 25.4 | 25.3 | 23.8 | 23.2 | 24.3 | 1308.19 |

**Supplementary Table 2**. Primary and secondary outcome means by trial arm and overall. Trial arms involve repositioning by: F (foods only), R (restaurants only), FR (foods and restaurants), and FRP (foods and restaurants plus price).

|  | Control | F | R | FR | FRP | Total |
| --- | --- | --- | --- | --- | --- | --- |
| **Mean Energy Selected** | 1382.28 | 1297.28 | 1220.77 | 1175.16 | 1266.28 | 1269.25 |
| **Total Price** | 14.91 | 14.11 | 14.12 | 13.78 | 16.25 | 14.63 |
| **Average Energy in Restaurant Mains** | 895.62 | 897.32 | 770.02 | 771.25 | 821.28 | 831.96 |
| **Total Energy in Standardised Serving of Selected Items** | 1404.68 | 1318.77 | 1239.13 | 1195.19 | 1291.28 | 1290.70 |

## Primary Analysis

Unless otherwise stated, all regression models use HC3 standard errors

*indicates B-H adjusted p-value for 10 comparisons

**Supplementary Table 3**. Primary analysis regression models using gamma regression.

| Term |  | Estimate | Standard Error | P-value |
| --- | --- | --- | --- | --- |
|  | (Intercept) | 7.14 | 0.05 | <0.001 |
| Treatment Group | Foods only | -0.07 | 0.02 | <0.001* |
| (Reference = Control) | Restaurants only | -0.12 | 0.02 | <0.001* |
|  | Foods and restaurants | -0.16 | 0.02 | <0.001* |
|  | Foods and restaurants plus price | -0.09 | 0.02 | <0.001* |
| Age Group | 25 to 54 | 0.04 | 0.02 | 0.029 |
| (Reference = 18 - 24) | 55 and over | 0.05 | 0.02 | 0.014 |
| Gender | Male | 0.00 | 0.01 | 0.836 |
| (Reference = Female) | Other gender | 0.05 | 0.05 | 0.307 |
| Income (Ref = < £30k) | £30,000 and over | 0.02 | 0.01 | 0.080 |
| Location | Midlands | 0.00 | 0.02 | 0.943 |
| (Reference = London) | North | -0.02 | 0.02 | 0.334 |
|  | South and East | 0.01 | 0.02 | 0.785 |
|  | Wales, Scotland & Northern Ireland | -0.03 | 0.02 | 0.246 |
| Education | Less than high school | 0.01 | 0.04 | 0.801 |
| (Reference = High school completed | None of the above | -0.01 | 0.06 | 0.870 |
|  | University degree | -0.01 | 0.01 | 0.231 |
| BMI | Obese | 0.07 | 0.01 | <0.001 |
| (Reference = Healthy) | Overweight | 0.04 | 0.01 | 0.006 |
|  | Underweight | 0.00 | 0.04 | 0.898 |
| Socioeconomic Position | High | -0.02 | 0.02 | 0.429 |
| (Reference = Low) | Medium | -0.01 | 0.02 | 0.645 |
| Ethnicity | Black | 0.01 | 0.04 | 0.892 |
| (Reference = Asian) | Mixed | 0.03 | 0.04 | 0.452 |
|  | Other | 0.03 | 0.07 | 0.654 |
|  | White | 0.04 | 0.03 | 0.157 |
| Residence Area | Suburban | -0.03 | 0.01 | 0.051 |
| (Reference = Rural) | Urban | -0.06 | 0.02 | <0.001 |
| Order Frequency | A few times a week | 0.00 | 0.02 | 0.803 |
| (Reference = Less than once a month) | Every day | -0.22 | 0.05 | <0.001 |
|  | Once a month | 0.03 | 0.02 | 0.032 |
|  | Once a week | 0.01 | 0.02 | 0.407 |
| Time of Day | Afternoon – Lunch | 0.03 | 0.02 | 0.129 |
| (Reference = Morning - Breakfast) | Evening – Dinner | 0.05 | 0.02 | 0.012 |
|  | Night | 0.04 | 0.02 | 0.030 |
| Day of the Week | Monday | 0.02 | 0.04 | 0.710 |
| (Reference = Sunday) | Tuesday | -0.01 | 0.02 | 0.561 |
|  | Wednesday | -0.01 | 0.02 | 0.540 |
|  | Thursday | -0.01 | 0.02 | 0.694 |
|  | Friday | 0.04 | 0.03 | 0.227 |
|  | Saturday | -0.04 | 0.05 | 0.359 |

**Supplementary Table 4**. Primary analysis robustness check using OLS regression

| Term |  | Estimate | Standard Error | P-value |
| --- | --- | --- | --- | --- |
|  | (Intercept) | 1258.17 | 63.87 | <0.001 |
| Treatment Group | Foods only | -87.15 | 22.17 | <0.001* |
| (Reference = Control) | Restaurants only | -159.01 | 21.61 | <0.001* |
|  | Foods and restaurants | -208.58 | 21.85 | <0.001* |
|  | Foods and restaurants plus price | -114.71 | 22.21 | <0.001* |
| Age Group | 25 to 54 | 49.81 | 21.31 | 0.019 |
| (Reference = 18 - 24) | 55 and over | 66.58 | 26.31 | 0.011 |
| Gender | Male | 6.33 | 14.12 | 0.654 |
| (Reference = Female) | Other gender | 54.54 | 61.54 | 0.375 |
| Income (Ref = < £30k) | £30,000 and over | 26.38 | 14.98 | 0.078 |
| Location | Midlands | -3.61 | 27.04 | 0.894 |
| (Reference = London) | North | -22.52 | 25.46 | 0.376 |
|  | South and East | 6.48 | 25.61 | 0.800 |
|  | Wales, Scotland and Northern Ireland | -32.32 | 28.42 | 0.256 |
| Education | Less than high school | 14.61 | 48.45 | 0.763 |
| (Reference = High school completed | None of the above | -15.16 | 75.10 | 0.840 |
|  | University degree | -16.10 | 15.38 | 0.295 |
| BMI | Obese | 88.65 | 17.82 | <0.001 |
| (Reference = Healthy) | Overweight | 46.50 | 16.52 | 0.005 |
|  | Underweight | -3.46 | 44.42 | 0.938 |
| Socioeconomic Position | High | -20.75 | 29.34 | 0.480 |
| (Reference = Low) | Medium | -12.08 | 26.31 | 0.646 |
| Ethnicity | Black | 11.29 | 46.68 | 0.809 |
| (Reference = Asian) | Mixed | 41.83 | 53.72 | 0.436 |
|  | Other | 42.04 | 91.43 | 0.646 |
|  | White | 53.86 | 31.53 | 0.088 |
| Residence Area | Suburban | -36.26 | 18.53 | 0.050 |
| (Reference = Rural) | Urban | -73.08 | 20.96 | <0.001 |
| Order Frequency | A few times a week | 9.51 | 24.29 | 0.695 |
| (Reference = Less than once a month) | Every day | -239.08 | 53.58 | <0.001 |
|  | Once a month | 41.85 | 19.62 | 0.033 |
|  | Once a week | 16.50 | 19.14 | 0.389 |
| Time of Day | Afternoon - Lunch | 39.87 | 24.92 | 0.110 |
| (Reference = Morning - Breakfast) | Evening - Dinner | 64.16 | 24.87 | 0.010 |
|  | Night | 56.06 | 25.18 | 0.026 |
| Day of the Week | Monday | 20.87 | 54.92 | 0.704 |
| (Reference = Sunday) | Tuesday | -13.68 | 28.60 | 0.633 |
|  | Wednesday | -15.41 | 27.77 | 0.579 |
|  | Thursday | -12.40 | 29.85 | 0.678 |
|  | Friday | 50.47 | 44.43 | 0.256 |
|  | Saturday | -52.86 | 56.94 | 0.353 |

## Secondary Analyses

**Supplementary Table 5**. Regression analysis of energy density

| Term |  | Estimate | Standard Error | P-value |
| --- | --- | --- | --- | --- |
|  | (Intercept) | 481.07 | 25.95 | <0.001 |
| Treatment Group | Foods only | -15.44 | 8.86 | 0.203* |
| (Reference = Control) | Restaurants only | -49.04 | 8.74 | <0.001* |
|  | Foods and restaurants | -71.48 | 8.91 | <0.001* |
|  | Foods and restaurants plus price | -16.11 | 9.00 | 0.203* |
| Age Group | 25 to 54 | 33.43 | 7.99 | <0.001 |
| (Reference = 18 - 24) | 55 and over | 47.88 | 10.45 | <0.001 |
| Gender | Male | 8.76 | 5.85 | 0.134 |
| (Reference = Female) | Other gender | -45.98 | 16.67 | 0.006 |
| Income  (Ref = < £30k) | £30,000 and over | 9.31 | 6.21 | 0.134 |
| Location | Midlands | 7.65 | 10.66 | 0.473 |
| (Reference = London) | North | 13.38 | 10.09 | 0.185 |
|  | South and East | -7.01 | 10.05 | 0.485 |
|  | Wales, Scotland and Northern Ireland | -3.13 | 11.25 | 0.781 |
| Education | Less than high school | -15.28 | 18.00 | 0.396 |
| (Reference = High school completed | None of the above | -16.02 | 31.63 | 0.612 |
|  | University degree | 4.08 | 6.37 | 0.521 |
| BMI | Obese | 4.59 | 7.34 | 0.532 |
| (Reference = Healthy) | Overweight | 4.71 | 6.84 | 0.491 |
|  | Underweight | 3.57 | 16.77 | 0.832 |
| Socioeconomic Position | High | -14.88 | 12.68 | 0.241 |
| (Reference = Low) | Medium | -15.85 | 11.62 | 0.173 |
| Ethnicity | Black | -40.86 | 16.23 | 0.012 |
| (Reference = Asian) | Mixed | -12.35 | 19.66 | 0.530 |
|  | Other | 80.00 | 43.80 | 0.068 |
|  | White | 29.14 | 12.41 | 0.019 |
| Residence Area | Suburban | 10.17 | 7.81 | 0.193 |
| (Reference = Rural) | Urban | -5.73 | 8.46 | 0.498 |
| Order Frequency | A few times a week | -78.09 | 9.68 | <0.001 |
| (Reference = Less than once a month) | Every day | -137.10 | 20.14 | <0.001 |
|  | Once a month | -16.21 | 8.91 | 0.069 |
|  | Once a week | -43.86 | 8.78 | <0.001 |
| Time of Day | Afternoon - Lunch | 9.49 | 10.94 | 0.386 |
| (Reference = Morning - Breakfast) | Evening - Dinner | -0.66 | 10.73 | 0.951 |
|  | Night | -5.08 | 10.93 | 0.642 |
| Day of the Week | Monday | 20.31 | 24.61 | 0.409 |
| (Reference = Sunday) | Tuesday | 6.38 | 12.31 | 0.604 |
|  | Wednesday | 7.56 | 12.04 | 0.530 |
|  | Thursday | 1.73 | 12.65 | 0.891 |
|  | Friday | 34.99 | 20.69 | 0.091 |
|  | Saturday | -1.20 | 25.23 | 0.962 |

**Supplementary Table 6**. Regression analysis of mean calories of standardised servings of selected items.

| Term |  | Estimate | Standard Error | P-value |
| --- | --- | --- | --- | --- |
|  | (Intercept) | 861.45 | 30.82 | <0.001 |
| Treatment Group | Foods only | -1.51 | 10.60 | >0.999* |
| (Reference = Control) | Restaurants only | -126.44 | 10.53 | <0.001* |
|  | Foods and restaurants | -125.52 | 10.88 | <0.001* |
|  | Foods and restaurants plus price | -74.44 | 10.48 | <0.001* |
| Age Group | 25 to 54 | 24.98 | 9.85 | 0.011 |
| (Reference = 18 - 24) | 55 and over | 86.81 | 12.70 | <0.001 |
| Gender | Male | -19.29 | 7.03 | 0.006 |
| (Reference = Female) | Other gender | -64.11 | 26.90 | 0.017 |
| Income  (Ref = < £30k) | £30,000 and over | 5.56 | 7.49 | 0.458 |
| Location | Midlands | 27.37 | 13.01 | 0.035 |
| (Reference = London) | North | 33.06 | 12.28 | 0.007 |
|  | South and East | 14.96 | 12.18 | 0.219 |
|  | Wales, Scotland and Northern Ireland | 17.29 | 13.63 | 0.205 |
| Education | Less than high school | -5.14 | 20.87 | 0.806 |
| (Reference = High school completed | None of the above | -29.40 | 34.86 | 0.399 |
|  | University degree | -4.79 | 7.66 | 0.532 |
| BMI | Obese | 29.23 | 8.90 | 0.001 |
| (Reference = Healthy) | Overweight | 12.14 | 8.17 | 0.137 |
|  | Underweight | -27.30 | 18.15 | 0.133 |
| Socioeconomic Position | High | -1.64 | 14.20 | 0.908 |
| (Reference = Low) | Medium | -1.59 | 12.72 | 0.900 |
| Ethnicity | Black | -1.40 | 21.31 | 0.948 |
| (Reference = Asian) | Mixed | -17.67 | 25.25 | 0.484 |
|  | Other | -12.64 | 43.48 | 0.771 |
|  | White | 26.50 | 15.40 | 0.085 |
| Residence Area | Suburban | 6.17 | 9.17 | 0.501 |
| (Reference = Rural) | Urban | -3.44 | 10.13 | 0.734 |
| Order Frequency | A few times a week | -73.13 | 11.67 | <0.001 |
| (Reference = Less than once a month) | Every day | -120.78 | 25.28 | <0.001 |
|  | Once a month | -3.28 | 10.32 | 0.751 |
|  | Once a week | -38.46 | 10.14 | <0.001 |
| Time of Day | Afternoon - Lunch | -5.06 | 12.50 | 0.686 |
| (Reference = Morning - Breakfast) | Evening - Dinner | -1.46 | 12.43 | 0.907 |
|  | Night | -7.35 | 12.50 | 0.556 |
| Day of the Week | Monday | 8.47 | 27.38 | 0.757 |
| (Reference = Sunday) | Tuesday | -14.54 | 14.21 | 0.306 |
|  | Wednesday | -9.47 | 13.97 | 0.498 |
|  | Thursday | -11.65 | 14.80 | 0.431 |
|  | Friday | 1.98 | 22.58 | 0.930 |
|  | Saturday | -34.61 | 27.40 | 0.207 |

**Supplementary Table 7**. Regression analysis for price of items placed in baskets

| Term |  | Estimate | Standard Error | P-value |
| --- | --- | --- | --- | --- |
|  | (Intercept) | 2.71 | 0.05 | <0.001 |
| Treatment Group | Foods only | -0.05 | 0.02 | 0.013* |
| (Reference = Control) | Restaurants only | -0.05 | 0.02 | 0.013* |
|  | Foods and restaurants | -0.08 | 0.02 | <0.001* |
|  | Foods and restaurants plus price | 0.09 | 0.02 | <0.001* |
| Age Group | 25 to 54 | -0.02 | 0.02 | 0.379 |
| (Reference = 18 - 24) | 55 and over | -0.04 | 0.02 | 0.076 |
| Gender | Male | 0.00 | 0.01 | 0.868 |
| (Reference = Female) | Other gender | 0.11 | 0.05 | 0.033 |
| Income (Ref = < £30k) | £30,000 and over | 0.02 | 0.01 | 0.170 |
| Location | Midlands | -0.01 | 0.02 | 0.624 |
| (Reference = London) | North | -0.01 | 0.02 | 0.796 |
|  | South and East | -0.01 | 0.02 | 0.589 |
|  | Wales, Scotland and Northern Ireland | -0.02 | 0.02 | 0.305 |
| Education | Less than high school | -0.01 | 0.04 | 0.845 |
| (Reference = High school completed | None of the above | 0.05 | 0.07 | 0.435 |
|  | University degree | 0.03 | 0.01 | 0.011 |
| BMI | Obese | 0.06 | 0.01 | <0.001 |
| (Reference = Healthy) | Overweight | 0.05 | 0.01 | <0.001 |
|  | Underweight | 0.01 | 0.04 | 0.795 |
| Socioeconomic Position | High | 0.05 | 0.02 | 0.035 |
| (Reference = Low) | Medium | 0.03 | 0.02 | 0.147 |
| Ethnicity | Black | -0.04 | 0.04 | 0.354 |
| (Reference = Asian) | Mixed | -0.03 | 0.05 | 0.486 |
|  | Other | 0.01 | 0.09 | 0.887 |
|  | White | -0.05 | 0.03 | 0.094 |
| Residence Area | Suburban | -0.06 | 0.01 | <0.001 |
| (Reference = Rural) | Urban | -0.08 | 0.02 | <0.001 |
| Order Frequency | A few times a week | 0.07 | 0.02 | 0.001 |
| (Reference = Less than once a month) | Every day | -0.03 | 0.06 | 0.593 |
|  | Once a month | 0.03 | 0.02 | 0.028 |
|  | Once a week | 0.05 | 0.02 | 0.002 |
| Time of Day | Afternoon - Lunch | -0.01 | 0.02 | 0.686 |
| (Reference = Morning - Breakfast) | Evening - Dinner | 0.06 | 0.02 | 0.006 |
|  | Night | 0.04 | 0.02 | 0.065 |
| Day of the Week | Monday | 0.00 | 0.04 | 0.994 |
| (Reference = Sunday) | Tuesday | -0.05 | 0.02 | 0.055 |
|  | Wednesday | -0.05 | 0.02 | 0.026 |
|  | Thursday | -0.03 | 0.03 | 0.305 |
|  | Friday | 0.00 | 0.04 | 0.899 |
|  | Saturday | -0.02 | 0.05 | 0.708 |

## Demographic Analyses

All models use HC3 robust standard errors

*indicates B-H adjusted p-value for 10 comparisons

**Supplementary Table 8**. Main effects and interaction effects coefficients from BMI interaction model.

| Term |  | Estimate | Standard Error | P-value |
| --- | --- | --- | --- | --- |
|  | (Intercept) | 1343.89 | 65.44 | <0.001 |
| Treatment Group | Foods only | -106.13 | 29.74 | 0.001* |
| (Reference = Control) | Restaurants only | -181.14 | 29.62 | <0.001* |
|  | Foods and restaurants | -232.73 | 29.51 | <0.001* |
|  | Foods and restaurants plus price | -136.60 | 30.37 | <0.001* |
| BMI Category (Ref = Overweight/Obese) | Underweight/Healthy | -102.29 | 32.11 | 0.001 |
| Interaction Effects | Foods only: Underweight/Healthy | 41.04 | 44.55 | 0.893* |
| (Reference = Overweight/Obese) | Restaurants only: Underweight/Healthy | 46.92 | 43.35 | 0.893* |
|  | Foods and restaurants: Underweight/Healthy | 52.92 | 44.02 | 0.893* |
|  | Foods and restaurants plus price: Underweight/Healthy | 46.39 | 44.62 | 0.893* |

**Supplementary Table 9**. Main effects and interaction effects coefficients from gender interaction model.

| Term |  | Estimate | Standard Error | P-value |
| --- | --- | --- | --- | --- |
|  | (Intercept) | 1278.92 | 65.45 | <0.001 |
| Treatment Group | Foods only | -75.93 | 32.73 | 0.051* |
| (Reference = Control) | Restaurants only | -187.47 | 31.19 | <0.001* |
|  | Foods and restaurants | -273.22 | 31.62 | <0.001* |
|  | Foods and restaurants plus price | -175.33 | 32.33 | <0.001* |
| Gender Category  (Ref = Male) | Female | -54.70 | 32.16 | 0.089 |
| Interaction Effects | Foods only: Female | -25.03 | 44.64 | >0.999* |
| (Reference = Male | Restaurants only: Female | 48.19 | 43.54 | 0.895* |
|  | Foods and restaurants: Female | 109.29 | 43.87 | 0.063* |
|  | Foods and restaurants plus price: Female | 112.40 | 44.77 | 0.063* |

**Supplementary Table 10**. Main effects and interaction effects coefficients from socioeconomic position interaction model.

| Term |  | Estimate | Standard Error | P-value |
| --- | --- | --- | --- | --- |
|  | (Intercept) | 1224.03 | 60.08 | <0.001 |
| Treatment Group | Foods only | -77.16 | 29.24 | 0.028* |
| (Reference = Control) | Restaurants only | -117.77 | 28.50 | <0.001* |
|  | Foods and restaurants | -178.39 | 28.48 | <0.001* |
|  | Foods and restaurants plus price | -63.64 | 28.97 | 0.070* |
| SEP Category | Low | 37.68 | 60.72 | 0.535 |
| (Reference = Medium) | High | 58.57 | 34.54 | 0.090 |
| Interaction Effects | Foods only: Low | -33.92 | 79.70 | >0.999* |
| (Reference = Medium) | Restaurants only: Low | -20.46 | 83.24 | >0.999* |
|  | Foods and restaurants: Low | -64.24 | 83.47 | >0.999* |
|  | Foods and restaurants plus price: Low | -0.60 | 85.66 | >0.999* |
| Interaction Effects | Foods only: High | -16.34 | 48.11 | >0.999* |
| (Reference = Medium) | Restaurants only: High | -110.79 | 46.46 | 0.086* |
|  | Foods and restaurants: High | -68.64 | 47.33 | 0.490* |
|  | Foods and restaurants plus price: High | -147.01 | 47.97 | 0.022* |

**Supplementary Table 11**. Main effects and interaction effects coefficients from interaction model on frequency of using food delivery platforms

| Term |  | Estimate | Standard Error | P-value |
| --- | --- | --- | --- | --- |
|  | (Intercept) | 1289.11 | 64.13 | <0.001 |
| Treatment Group | Foods only | -101.44 | 32.25 | 0.004* |
| (Reference = Control) | Restaurants only | -222.65 | 30.20 | <0.001* |
|  | Foods and restaurants | -243.77 | 30.92 | <0.001* |
|  | Foods and restaurants plus price | -159.61 | 31.30 | <0.001* |
| Delivery Platform Use  (Ref = Once a week or more) | Less than once a week | -46.97 | 32.16 | 0.153 |
| Interaction Effects | Foods only: Less than once a week | 32.52 | 44.32 | >0.999* |
| (Reference = Once a week or more) | Restaurants only: Less than once a week | 133.41 | 43.30 | 0.021* |
|  | Foods and restaurants: Less than once a week | 75.67 | 43.70 | 0.278* |
|  | Foods and restaurants plus price: Less than once a week | 90.02 | 44.50 | 0.216* |

**Supplementary Table 12**. Main effects and interaction effects coefficients from interaction model on device used for the survey.

| Term |  | Estimate | Standard Error | P-value |
| --- | --- | --- | --- | --- |
|  | (Intercept) | 1199.10 | 70.81 | <0.001 |
| Treatment Group | Foods only | -78.36 | 46.90 | 0.475* |
| (Reference = Control) | Restaurants only | 3.38 | 50.87 | >0.999* |
|  | Foods and restaurants | -95.54 | 47.91 | 0.453* |
|  | Foods and restaurants plus price | -52.15 | 49.08 | 0.960* |
| Survey Method  (Ref = Desktop) | Mobile Phone | 71.99 | 40.03 | 0.071 |
| Interaction Effects | Foods only: Mobile Phone | -8.50 | 53.18 | >0.999* |
| (Reference = Desktop) | Restaurants only: Mobile Phone | -199.13 | 56.13 | 0.003* |
|  | Foods and restaurants: Mobile Phone | -139.37 | 53.80 | 0.046* |
|  | Foods and restaurants plus price: Mobile Phone | -76.52 | 54.97 | 0.547* |
