# Supplementary material for: The impact of altering restaurant and menu option position on food selected from an experimental food delivery platform: a randomised controlled trial
Source: Int J Behav Nutr Phys Act. 2023 May 19;20:60. doi: 10.1186/s12966-023-01456-8 (PMC10197857; doi:10.1186/s12966-023-01456-8)
Supplement: Supplementary file 2 — Supplementary Material 2 [file 12966_2023_1456_MOESM2_ESM.docx]

EDITOR COMMENTS:

Comments to the author (if any):

Associate Editor: Thank you for your thorough review. Please address the suggested edits by reviewers.

REVIEWER #1 COMMENTS:

Reviewer #1: Thank you for your extensive and comprehensive responses to the review process. Your revised paper is much better. There is just one, very small, thing I would ask that you do and that is where you introduce the control condition in the paper (page 7 line 138), please add one sentence to indicate that random is only within each category (appetisers, mains, etc). You state this clearly later, but having it here may save future readers' confusion.

*Thank you for your comment. Lines 129-31 now read:* Food items were randomly ordered within food categories, but the order in which food categories appear was not randomised (e.g. starters presented first, followed by mains, etc.)

REVIEWER #2 COMMENTS:

Reviewer #2: The authors have thoroughly address all comments, I just have one outstanding comment -

The rationale for gamma regression was that there was an expected rightward skew - was this tested to confirm with the actual data? This would be preferable to confirm rather than base on expected patterns

*We have now added to the methods, and lines 236-7 read: “*Data were assessed visually to consider the skew of outcome variables and confirm the appropriate model was applied”
